# Study of the Influence of the Crystallographic Orientation of Cassiterite Observed with Colloidal Probe Atomic Force Microscopy and its Implications for Hydrophobization by an Anionic Flotation Collector

**DOI:** 10.1021/acsomega.0c03980

**Published:** 2021-02-03

**Authors:** Haosheng Wu, Axel D. Renno, Yann Foucaud, Martin Rudolph

**Affiliations:** † Helmholtz-Zentrum Dresden-Rossendorf (HZDR), Helmholtz Institute Freiberg for Resource Technology (HIF), Chemnitzer Str. 40, Freiberg 09599, Germany; ‡ ICSM, Univ Montpellier, CEA, CNRS, ENSCM, Marcoule, Bagnols-sur-Cèze 30207, France

## Abstract

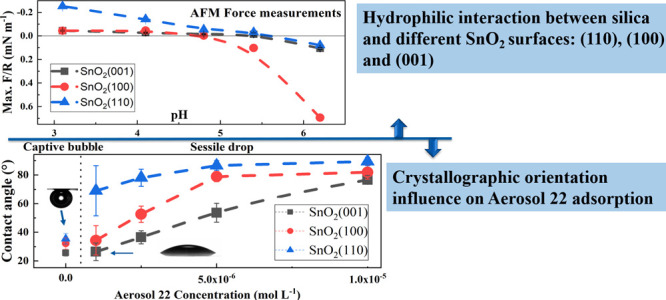

In this study, the physicochemical behaviors of the (110), (100), as well as (001) of SnO_2_ were investigated by using high-resolution direct force spectroscopy. The measurements were conducted between a silica sphere and sample surfaces in 10 mmol/L KCl between pH 3.1 and 6.2 using colloidal probe atomic force microscopy (cp-AFM-hydrophilic). Dissimilar interactions were detected on different-oriented surfaces. The pH values where the force switched from positive to negative can be clearly distinguished and be ordered as SnO_2_(100) < SnO_2_(001) ≈ SnO_2_(110). By fitting the force curves in the Derjaguin–Landau–Verwey–Overbeck theory framework, anisotropic surface potentials were computed between the three sample surfaces following a similar trend as force interaction. To study the implication of crystallographic orientation to surfactant adsorption, we used Aerosol 22 (sulfosuccinamate) as an anionic collector for cassiterite flotation to functionalize the different samples at pH 3. The contact angle measurements, the topography visualizations by AFM, and the force measurement using cp-AFM with hydrophobized spheres (cp-AFM-hydrophobized) have shown that Aerosol 22 was adsorbed on the sample surfaces inhomogeneously. The adsorption followed the range of SnO_2_(110) > SnO_2_(100) > SnO_2_(001) in the concentration from 1 × 10^–6^ to 1 × 10^–4^ mol/L.

## 1 Introduction

With the development of electronic devices, global tin demand has significantly and continuously increased over the past decades. Cassiterite (SnO_2_) is the most important source of tin worldwide.^1^ During all cassiterite extraction and concentration processes, from grinding to the various following processing stages,^2^ the generation of fine cassiterite particles (<100 μm) is unavoidable due to the inherent brittle nature of this mineral.^3^ Once they are generated, cassiterite fine particles are generally concentrated by the froth flotation method, which is based on bubble-particle heterocoagulation and fundamentally relies on the contrast of particle surface properties. The selective adsorption of surfactants, called flotation collectors, hydrophobizes the target mineral(s) and induces their subsequent recovery in the froth flotation.

In flotation, the mineral surface potential plays a crucial role in the adsorption of collector molecules at the liquid/solid interface,^4^ especially when electrostatic interaction controls the adsorption process, as is the case for oxidic minerals^5^ such as cassiterite. Moreover, tin ores are becoming more complex; that is, cassiterite is more often associated with minerals that display similar physicochemical properties. Therefore, understanding the influence of the cassiterite crystal structure on the adsorption of collectors is of paramount interest to improve flotation selectivity and efficiency. Over the past decades, colloidal force measurements for different crystallographic orientations of minerals have been intensively explored.^6−13^ For instance, the study of Gao and co-workers^14^ analyzed the interactions between silicon nitride cantilever tips and three scheelite (Ca[WO_4_]) cleavage surfaces by using high-resolution atomic force spectroscopy (AFM). They calculated the surface potential values by fitting the force data to the Derjaguin–Landau–Verwey–Overbeck (DLVO) theory and demonstrated that the electrostatic potential for (001) surface was only slightly affected by pH. In contrast, the surface potential for both (112) and (101) increased with pH. Besides, Bullard and Cima^15^ have also used AFM to measure interactions between a silica sphere and rutile (TiO_2_) (110), (100), as well as (001) surfaces in multiple solution conditions over the broad range of pH values. They exhibited a strong anisotropic behavior in the measured surface potential. Nevertheless, Kallay and Preočanin,^16^ who used more direct experimental methods to study individual crystal planes of hematite (Fe_2_O_3_) found no significant difference in surface potential between the (012), (10–2), (113), and (11–3) surfaces in a low ionic strength solution. To the best of our knowledge, no previous study has investigated the surface potential of different cleavage surfaces of cassiterite. Furthermore, although the adsorption of different types of collector molecules on the (110) cassiterite surface was investigated by molecular dynamic simulations,^17−19^ very few studies explored the difference(s) between the different cleavage surfaces of cassiterite in terms of collector adsorption.^18^

In this study, we investigated the differences in terms of surface potential between the three main cleavage planes of cassiterite, namely the (110), (100), and (001) surfaces. The interaction forces were acquired using colloidal probe force spectroscopy with unfunctionalized silica spheres as colloidal probes (cp-AFM-hydrophilic). Furthermore, contact angle measurements were combined with cp-AFM using hydrophobized spheres (cp-AFM-hydrophobized) to assess the wettability of the three aforementioned cassiterite planes before and after the adsorption of the anionic Aerosol 22, a sulfosuccinamate that is one of the most important collectors for cassiterite flotation.^20^

## 2 Results

### 2.1 Surface Characterization of Cassiterite Surfaces

The square roughness *R*_q_ as well as the arithmetic mean roughness *R*_a_ in the scan region of both 2 μm × 2 μm and 8 μm × 8 μm are summarized in Table 1. The measured roughness values in the same scan region vary within a narrow range, while the roughness values in 8 μm × 8 μm are, however, much larger than the values in 2 μm × 2 μm. It was caused by tiny scratches on a larger scale (for AFM topographic images, please refer to Appendix). The strips might be caused by sample polishing during sample embedding in epoxy as well as sample cleaning steps, in which they might be further created during sample polishing. The roughness values before as well as after the sample cleaning steps were thus compared, and it was found that the measured roughness value variations do not depend on polishing. Furthermore, it is noted that the surface roughness, as such, is comparable between the different single crystals. Therefore, the scan region for the sensitive hydrophilic force measurement was chosen to be within 2 μm × 2 μm, while for hydrophobic force measurements within 8 μm × 8 μm.

**Table 1:** Roughness Defined as *R*_q_ (Root Mean Square of the Measured Height) and *R*_a_ (Arithmetic Mean Value of Filtered Roughness Profile) of Sample SnO_2_(110), SnO_2_(100), and SnO_2_(001) Measured in a Measuring Range of 8 μm × 8 μm and 2 μm × 2 μm

	*R*_q_ (nm) (8 μm × 8 μm)	*R*_q_ (nm) (2 μm × 2 μm)	*R*_a_ (nm) (8 μm × 8 μm)	R_a_ (nm) (2 μm × 2 μm)
SnO_2_(110)	0.91 ± 0.08	0.32 ± 0.07	0.65 ± 0.06	0.25 ± 0.05
SnO_2_(100)	1.12 ± 0.05	0.26 ± 0.04	0.78 ± 0.07	0.21 ± 0.04
SnO_2_(001)	0.80 ± 0.06	0.41 ± 0.06	0.58 ± 0.05	0.32 ± 0.04

### 2.2 Forces between Silica Sphere and SnO_2_ Surfaces

AFM force measurements were conducted to measure the interaction of the (110), (100), and (001) cassiterite surfaces with silica colloidal probe in 10 mmol/L KCl electrolyte at different pH values. The determination of zero force was taken where a very low and constant deflection of the cantilever was detected far away (600 nm) from sample surfaces.^21,22^ The determination of zero distance is chosen at which the cantilever deflection is starting to be linear to sample displacement,^21,23^ which indicates that the cantilever is in contact with the sample surface.

**Figure 1:**
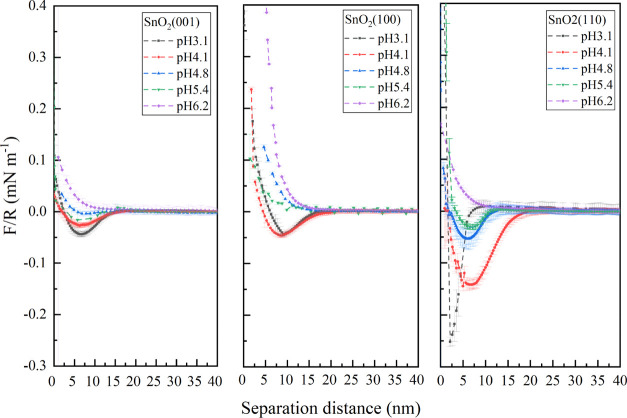
Averaged normalized force curves (*F*/*R*) in approach as a function of the separation distance between silica particles and the sample surface. The measurements were conducted in 10 mmol/L KCl solution at different pH (3.1, 4.1, 4.8, 5.4, and 6.2). The minus and plus error bars range represents the 95% confidence interval of 64 measurements.

On the same sample surface, the observed attractive as well as repulsive forces closer to the sample surface (<20 nm) kept stable in a certain range (∼±0.02 mN/m as can be seen in Figure 1). Measurements were performed with an approaching speed of 0.6–0.3 μm s^–1^, resulting in no detectable changes of the force curves, indicating that the detected short-range forces were close to a steady-state, that is hydrodynamic forces are assumed to be neglectable.

It should be noticed that the interaction between the silica and the sample surface appeared at different separation distances, and almost all force curves follow the trend of larger interaction starting at a longer separation distance. However, SnO_2_(110) measured at pH 3.1 is not following the trend. It might be caused by the thermal drift of cantilever^23^ and the deviation of the zero-distance determination (owing to the deviation of cantilever spring constant). However, it is also worth noticing that this 5-time more potent attractive force on SnO_2_(110) at pH 3.1 can be better explained under the constant potential boundary condition within the framework of DLVO theory.^24^ At constant potential, the surface with the same (but not identical) potential shows a stronger repulsive force at a large separation distance before the attractive force occurred; thus, it is also reasonable to include the possibility of charge regulation on (110) surface at pH 3.1 (more discussions are in Appendix).

As the force–distance relationship still contains uncertainty, more essential and reliable information comes from the measured force alone. The interaction force between the (100) cassiterite surface and silica sphere switches from negative to positive values in the pH range of 4.1–4.8, while this change happens in the range of 5.4–6.2 for the (110) surface. For the (001) cassiterite surface, the attractive force values stay relatively stable from pH 3.1 to 5.4 and change to positive values when the pH reaches 6.2. A clear distinction of the normalized forces can be seen between the different crystallographic orientations exposed on the surfaces. Moreover, the attractive interactions between the (110) surface and silica sphere are always the largest compared to the (100) and (001) surfaces at lower pH, while the repulsive forces are the lowest at higher pH. The pH values where the force switched from positive to negative can be distinguished and be ordered as SnO_2_(100) < SnO_2_(001) ≈ SnO_2_(110). A similar trend is also observed when fitting the force curves within the framework of DLVO interactions assuming constant charge, as shown in Appendix.

### 2.3 Surface Characterization of Functionalized Surfaces

**Figure 2:**
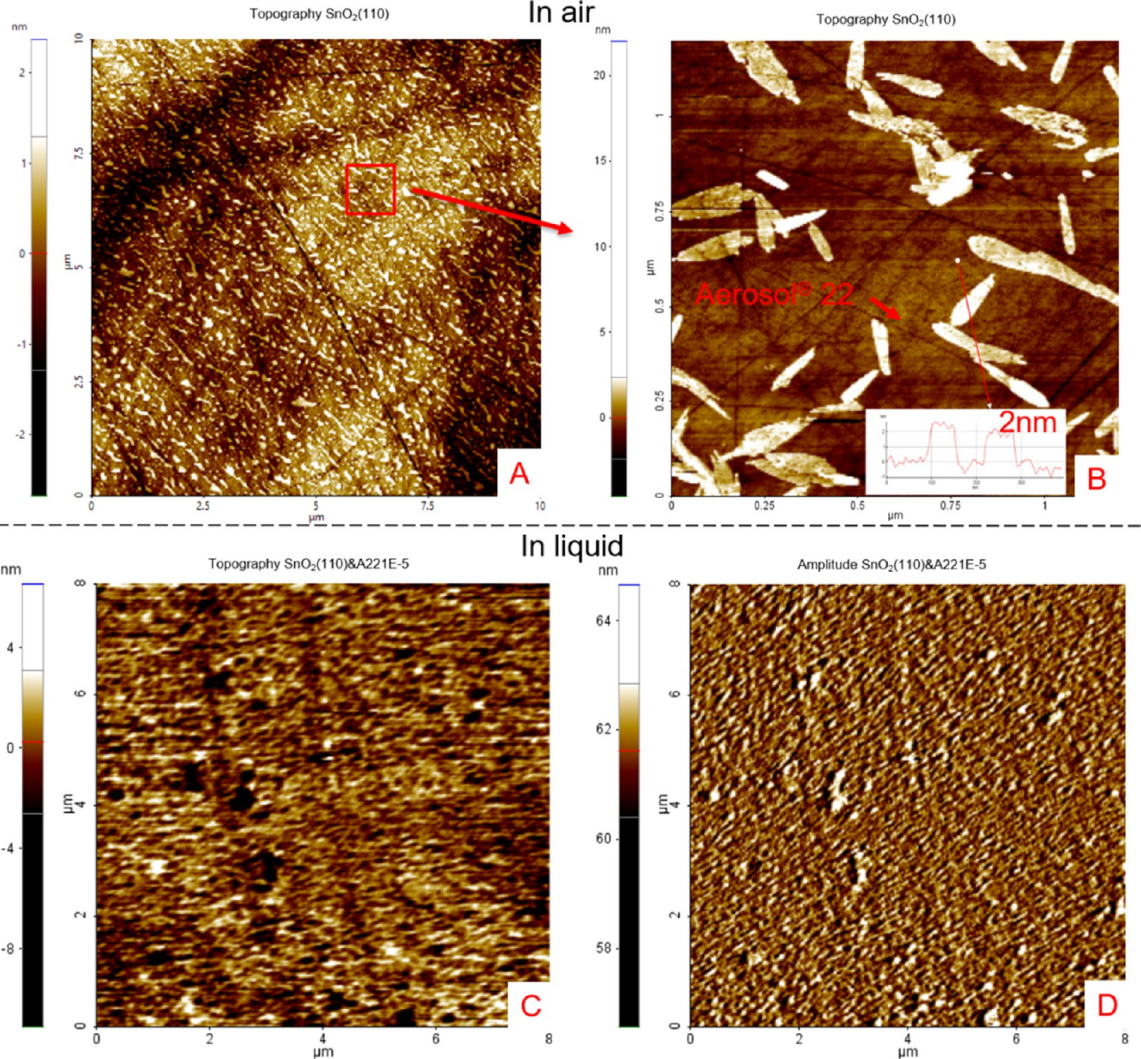
(A) Surface topographies of the functionalized (110) cassiterite surface in 10 × 10 μm. (B) One part from image (A) with cross-section profiles in 1.2 μm × 1.2 μm. (A,B) Sample was conditioned with 1 × 10^–4^ mol L^–1^ Aerosol 22 and measured in the air after the sample was blown dry with oxygen (following the same sample preparation for contact angle measurement); (C) Surface topographies of functionalized (110) cassiterite surface in 8 μm × 8 μm. (D) Amplitude image (error signal) of the image (C). (C,D) Sample was measured in 1 × 10^–5^ mol L^–1^ Aerosol 22 Solution at pH 3.

Figure 2 shows topography images of the (110) cassiterite surface conditioned in a 1 × 10^–5^ mol L^–1^ Aerosol 22 solution at pH 3. The resolution of topographic images taken in the liquid is relatively blurry. However, both the topographies in air and in liquid exhibit a significant number of patches assembled on the sample surfaces, which are most probably the adsorbed hemimicelles^25^ of Aerosol 22. The average height of the patches is around 2 nm, which is close to the height of the monolayer of Aerosol 22 (structure of Aerosol 22 as well as the topography images of Aerosol 22 on (100) and (001) surfaces are in Appendix). Their shape and distribution varied considerably. The adsorption of Aerosol 22 on the cassiterite surfaces can thus be described as inhomogeneous. Because of the variability in shape and distribution of the patches, no clear distinction was established between the composite Aerosol 22 layers on differently oriented cassiterite surfaces.

### 2.4 Forces between Hydrophobized Silica and Functionalized SnO_2_ Surfaces

**Figure 3:**
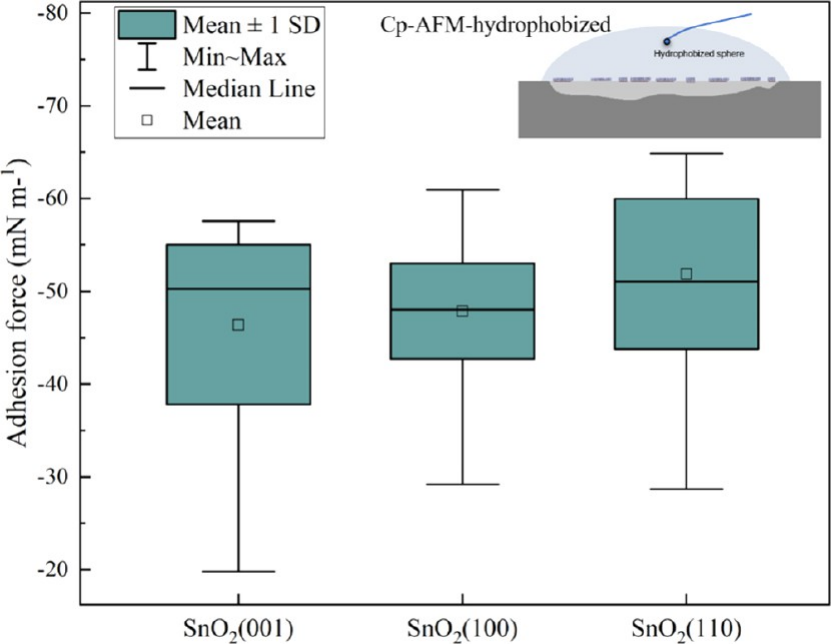
Normalized adhesion force distribution (mN m^–1^) of 328–492 force curves on each surface of SnO_2_(110), (100), and (001) in a solution with 1 × 10^–4^ mol L^–1^ Aerosol 22 at pH 3.1. The silanized silica colloid approached from a distance of 2 μm to the surface with a speed of 1 μm min^–1^ on each sample surface. The temperature of the liquid cell was set to 20 °C. S.D. stands for 1 standard deviation of the mean.

AFM was applied to analyze the hydrophobic interaction between the functionalized (110), (100), and (001) cassiterite surfaces and silanized silica sphere in 1 × 10^–4^ mol L^–1^ Aerosol 22 at pH 3.1. The adhesion forces, which are the pull-off forces (forces for colloid to detach from the sample surface) in the retrace curve, were analyzed. As shown in Figure 3, the adhesion forces varied significantly. Potential residuals of Aerosol 22 on the tip may be the cause of the capillary force differences. A two-sample *t*-test was thus carried out. The results show that at the 0.05 confidence interval, with a prior unequal variance *t*-test, the mean values of SnO_2_(001) and SnO_2_(100), as well as SnO_2_(100) and SnO_2_(110), are significantly different from each other; with a prior equal variance *t*-test, SnO_2_(001) and SnO_2_(110) is also significantly different from each other (data for the two-sample *T*-test are in Appendix). Thus, it is believed that the adsorption of Aerosol 22 is most effective on the (110) surface, followed by SnO_2_(100). The least adsorption of Aerosol 22 is on SnO_2_(001).

### 2.5 Contact Angle Measurements

**Figure 4:**
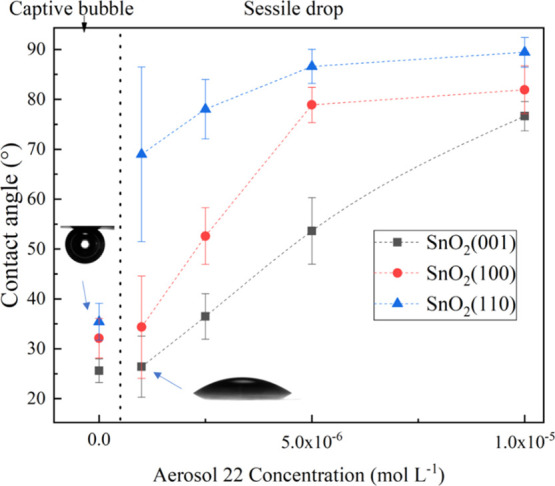
Contact angles measured on nonfunctionalized and functionalized (001), (100), and (110) cassiterite surfaces in water as a function of Aerosol 22 concentration (mol L^–1^). The error bars represent the 1 standard deviation calculated based on more than 16 measurement points.

The sessile drop method was used to qualitatively determine the adsorption ability of Aerosol 22 with respect to different crystallographic orientations. The static contact angles of water on (110), (100), and (001) cassiterite surfaces functionalized with 1 × 10^–6^ to 1 × 10^–5^ mol L^–1^ Aerosol 22 are presented in Figure 4. Furthermore, the captive bubble method was also conducted to measure the wettability of bare sample surfaces. The contact angles measured both on the bare as well as conditioned cassiterite (110) surface are consistently larger than on (100) and (001) surfaces, as shown in Figure 4.

The topography characterization seen in Figure 2 has shown a difference between the topographies of the adsorbed Aerosol 22 on the cassiterite surface in air and 1 × 10^–5^ mol L^–1^ Aerosol 22 solution. The abundance and distribution of adsorbed Aerosol 22 on the sample surface in solution are thus unnecessarily precisely the same compared to the adsorbed Aerosol 22 studied in sessile drop contact angle measurements. However, the measured contact angle is proportional to the coverage of Aerosol 22. A higher contact angle indicates stronger adsorption of Aerosol 22. As the measured contact angle follows the same range SnO_2_(110) > SnO_2_(100) > SnO_2_(001), the contact angle measurement results are in good agreement with the Cp-AFM-hydrophobized results.

## 3 Discussion

**Figure 5:**
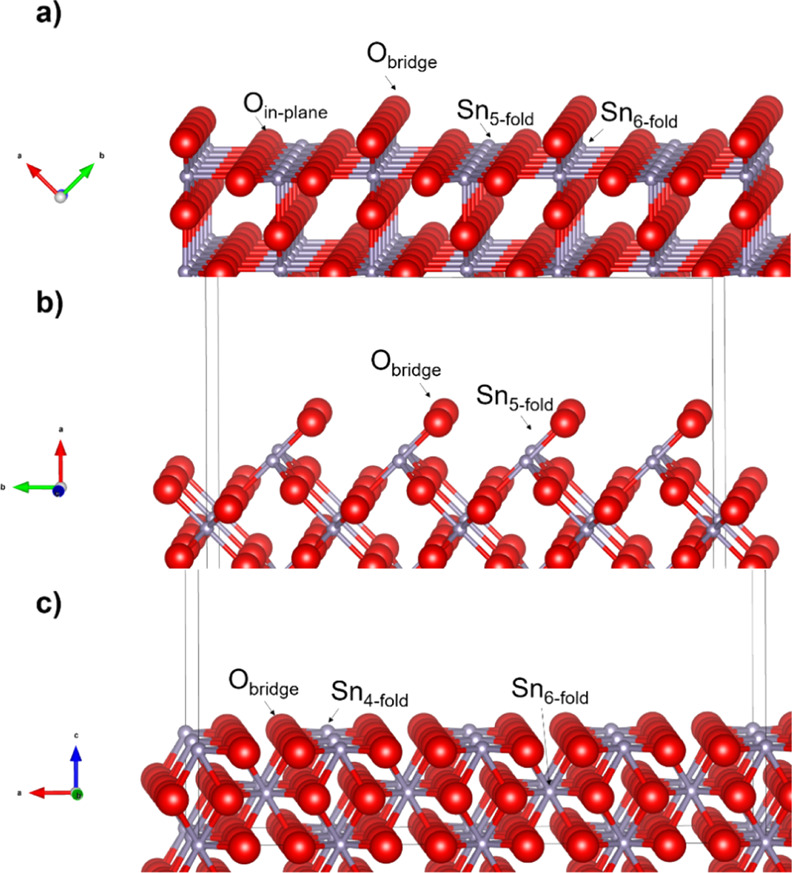
Ideal unrelaxed models of cassiterite surfaces (a) (110), (b) (100), and (c) (001), with Sn ions in light grey and O ions in red.

SnO_2_ has a ditetragonal dipyramidal crystallographic structure with a *P*4/*mnm* space group. This structure is characterized by two lattice parameters, *a* and *c* (*a* = 4.737 Å, *c* = 3.186 Å).^26^ In the SnO_2_ lattice, each Sn atom is coordinated to six O atoms, and each O atom is coordinated to three Sn atoms. In order to help the subsequent discussion of each surface, their main features are illustrated in Figure 5 based on theoretical calculation as well as X-ray diffraction (XRD) data.^27−29^

The unrelaxed (110) cassiterite surface includes the outermost plane of oxygen ions as the first atomic layer appears in rows along the [001] direction. These ions have one dangling bond and are called “bridging” (O_bridge_) ions. The second atomic layer includes 5-fold-coordinated (Sn_5-fold_) Sn cations, 6-fold-coordinated (Sn_6-fold_) Sn ions, and 3-fold-coordinated in-plane (O_in-plane_) O ions. The relaxation of the SnO_2_(110) surface studied by Batzill and co-workers^30^ results in downward relaxation of bridging oxygen ions with smaller downward shifts of Sn_5-fold_ and upward shifts of the Sn_6-fold_ ions.

Unlike the (110) surface, the outmost plane of the (100) surface includes only 2-fold-coordinated (O_bridge_) O ions followed by an atomic layer containing only 5-fold coordinated (Sn_5-fold_) Sn cations. Surface energy calculations have shown that the surface relaxations for (100) surface are very similar to those for the (110) surface, in that the bond length between Sn_5-fold_ and the O_bridge_ decreases, whereas the bond with sub-bridging O expands slightly. The movement of bridging O is outward.^27^

The outmost plane on the (001) cassiterite surface includes four-fold coordinated (Sn_4-fold_) Sn cations as well as two-fold coordinated O ions (O_bridge)_ followed by an atomic layer containing 6-fold (Sn_6-fold_) Sn ions and 3-fold O ions. The most striking feature on the relaxed (001) surface is the marked increase of corrugation, with four-fold Sn moving inward and six-fold surface Sn_6-fold_ moving outward.^27^

### 3.1 Force Curves Interpretation

**Figure 6:**
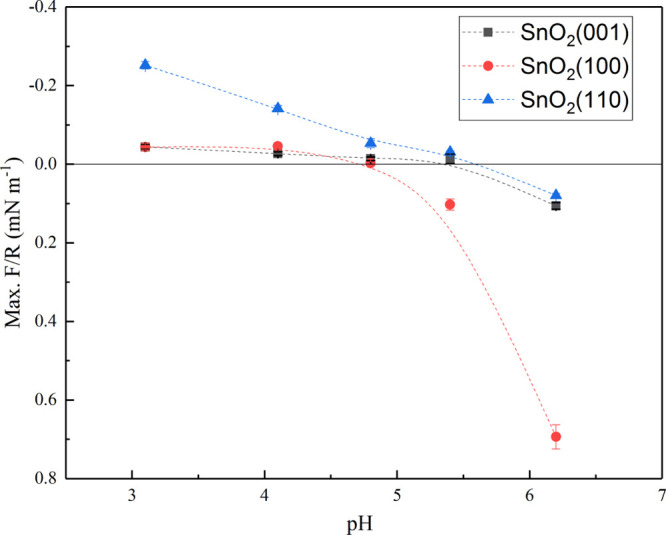
Comparison of normalized maximal forces *F*/*R* (mN m^–1^) between (110), (100), and (001) cassiterite surfaces and spherical silica tip in 1 mM KCl solution as a function of the solution pH. The minus and plus error bar range represents the 95% confidence interval of 64 measurements.

Figure 6 summarizes the most vital attractive forces resulting from the attractive interactions and the maximum repulsion forces in the noncontact phase resulting from the repulsive interaction and illustrates their dependence on pH. Force-changing signs can be well distinguished between the different orientations: SnO_2_(100) < SnO_2_(001) ≈ SnO_2_(110). Considering that the isoelectric point (IEP) of SiO_2_ is between pH = 2–3.4,^31−33^ one would expect that the IEP of the different SnO_2_ orientations is around pH = 4.5 for (100) and around pH = 5.5 for (110) and (001) (compared in Figure 6). The force–distance curves were fitted within the DLVO framework, that is an additive superposition of van der Waals and electric double layer forces. In short, the van der Waals interaction between silica and cassiterite across the water was evaluated from the full Lifshitz theory; the electric double layer interactions were treated within the linear regulation approximation.^24^ Although the latter theory is restricted to low potentials, it has the benefit of being able to account for charge regulation, constant potential, and constant charge interaction in a simple analytical formula (more details in Appendix). Compared to constant potential, the results show that the force curves can be better fitted under the boundary condition of constant charge. The calculated surface potential follows a similar trend, as shown in Figure 6: SnO_2_(100) < SnO_2_(001) ≈ SnO_2_(110) from pH = 4.1–6.2.

However, for the best-fitted force curves, the computed silica surface potentials at the same condition have considerable deviations by themselves. The uncertainty of the surface potential of the silica influences the results massively. For a precise quantification of the surface potential of the sample surfaces, independent experimental inputs (zeta potentials of both silica and cassiterite samples) are thus needed to guide the fit parameters. Unfortunately, based on the current experimental data, we cannot precisely quantify the surface potential for the different SnO_2_ orientations at each pH and locate their IEP. Nevertheless, the very fact that the pH with the force-changing sign depends on the crystallographic orientation implies a difference in surface properties. The results of the DLVO fitting further imply that under the same electrolyte condition, the three sample surfaces have anisotropic surface potentials (more details in Appendix).

### 3.2 Crystallographic Orientation Influence on Cassiterite Surface Hydration

Calatayud et al.^34^ pointed out that molecular adsorption on an oxide surface can be understood as an acid–base interaction. Given the lattice constants quoted above, the calculated cationic densities and the calculated broken bond densities for the unrelaxed surfaces are shown in Table 2.

**Table 2:** Comparison of Broken Bond Density, Cation Density, and Average Coordination Number of Sn among the Three Cassiterite Surfaces

orientation	broken bond density (nm^–2^)	cationic density (nm^–2^)	average coordination number
(110)	9.37	4.69	5.5
(100)	13.25	6.63	5
(001)	17.83	4.46	4

**Figure 7:**
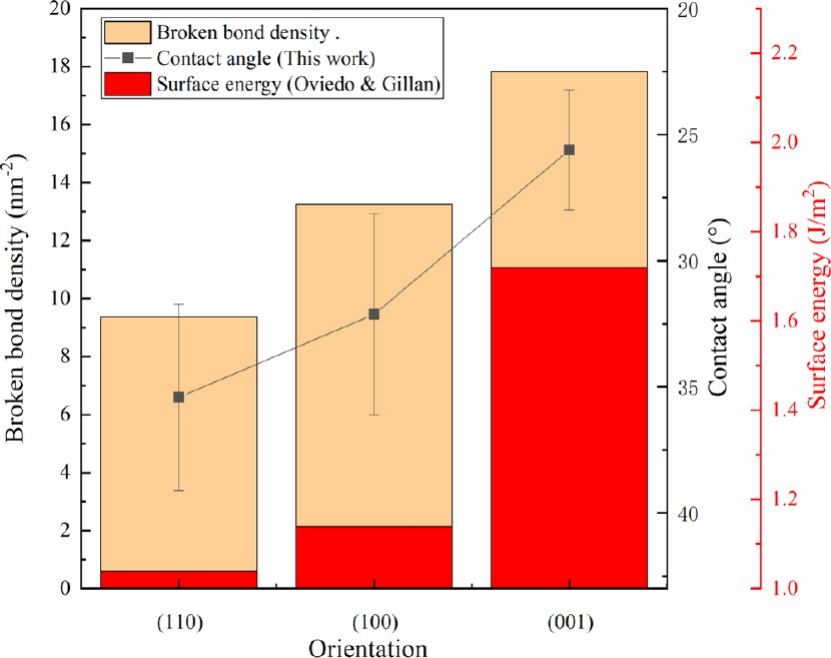
Calculated broken bond density (in nm^–2^) as a function of surface energies (in J/m^2^) for cassiterite calculated by Oviedo and Gillan^27^ as well as measured contact angles on the cassiterite bare surfaces in this study. Reprinted from Oviedo, J.; Gillan, M. J. Energetics and Structure of Stoichiometric SnO_2_ Surfaces Studied by First-Principles Calculations, *Surf. Sci.***2000**, *463*, 93, Copyright (2000), with permission from Elsevier.

Bullard and Cima^15^ also mentioned that based on the modern surface adsorption theory, the strength and extent of the cationic density of a given surface are believed to be a function of both the density as well as the electron affinity of available adsorption sites. The Sn cationic localities behave as Lewis acid sites for the adsorption of hydroxide ions.^35,36^ The O anionic positions, however, do not necessarily behave exclusively as Lewis basic sites. As shown in Table 2 as well as in Figure 7, the (100) surface has the highest cationic density and, after relaxation, a more substantial effect of Sn_5c_ cation. This would be a good reason to assume that compared to the (110) surface, the stronger and more effective adsorption of hydroxyl groups would be on the (100) surface at the same pH value. The (110) surface has a lower cationic density, and a lower electron affinity to Sn than the (100) surface, the adsorption of hydroxyl group would be thus less effective. Also, Evarestov et al.^37^ have mentioned that the “dissociative adsorption” of water is more favorable (by about 30 kJ/mol) for the (100) SnO_2_ surface, as in the case of the (110) surface. The absolute value of the adsorption energy of water on a (100) surface is lower than that on a (110) surface.^38^ Moreover, the simulations were all calculated with only molecular water. At lower pH at which a significant amount of H_3_O^+^ ion is available, its influence should be considered. However, no related study has yet been found.

**Table 3:** The Modes of AFM Measurements and their Corresponding Cantilever Type

mode	in air	in liquid
topographic imaging (tapping mode)	NCHR (320 kHz)	ConAl-G (7.5 kHz in liquid)
roughness quantification (tapping mode)		
force mapping (cp-AFM-hydrophilic)		All-in-one B (2.7 N/m)
force mapping(cp-AFM-hydrophobized)		TL-CONT (0.2 N/m)

The relative stable behavior of SnO_2_(001) in a broad range of pH observed from the Cp-AFM-hydrophilic measurements indicates a more complex system compared to SnO_2_(110) and SnO_2_(100). The results of SnO_2_(001) thus left a more open space for the discussion.

### 3.3 Crystallographic Orientation Influence on Cassiterite Surface Wettability

Wettability is defined as the ability of a liquid to maintain contact with a solid surface, and it is controlled by the balance between the intermolecular interactions of the adhesive type (for instance, liquid to solid) and cohesive type (for instance, liquid to liquid).^39^ As the liquid phase stays constant, it is reasonable to assume that the variations of the cationic and anionic sites on each studied cassiterite surface result in differences in adhesion interaction. In numerous simulation studies, the surface energy of different cassiterite surfaces in a vacuum has been calculated.^18,27,40^ By taking the abovementioned lattice parameter,^26^ the broken bond density is calculated and shown in Figure 7.

A direct link between the vacuum surface energy and the surface broken bond density can be seen in Figure 7, which was reported before.^41^ Moreover, the captive bubble measurement results in this study reveal the possibility of a direct relationship between the surface broken bond and wettability. The SnO_2_(110) has the lowest vacuum surface energy due to its lowest broken bond density, which might explain the lowest surface tension (largest contact angle) and its comparably lowest wettability.

### 3.4 Crystallographic Orientation Influence on Aerosol 22 Adsorption

Aerosol 22 is a complex anionic surfactant containing three carboxyl groups and a sulfonic group. Since its adsorption efficiency on cassiterite is strongly affected by pH,^42^ the electrostatic interactions should not be ignored. According to Arbiter, chemical interaction also played a dominant role.^43^ Only very little research has been conducted on the adsorption mechanism of this collector. The adsorption mechanism of one simple anionic surfactant oleate was studied by density functional theory (DFT) calculations.^18^ The results predict that the chemical interaction of oleate with the (110) cassiterite surface has in total less interaction energy than with the (100) cassiterite surface. However, the simulation also reported that there might be a shielding effect of the topmost oxygen layer on the (100) cassiterite surface that prevents the interaction. The oleate was thus predicted to have the most effective adsorption on the (110) surface. Even though different anionic surfactants have been used, the DFT calculation results match the experimental findings in this study. An assumption would be that the interaction energy and the potential spatial benefit of (110) cassiterite surface enrich the adsorption of Aerosol 22.

Moreover, as a higher attractive interaction was measured on the (110) cassiterite surface as compared to the (100) surface at pH 3, the electrokinetic behavior of SnO_2_ single crystals should be studied in detail. It should be stressed that neither the chemical interaction nor the electrostatic interaction of the collector on the three cassiterite surfaces can be interpreted thoroughly from the mentioned DFT calculation or the force difference, respectively. However, the foregoing discussion of different experimental approaches can enrich the understanding of this complex system.

**Figure 8:**
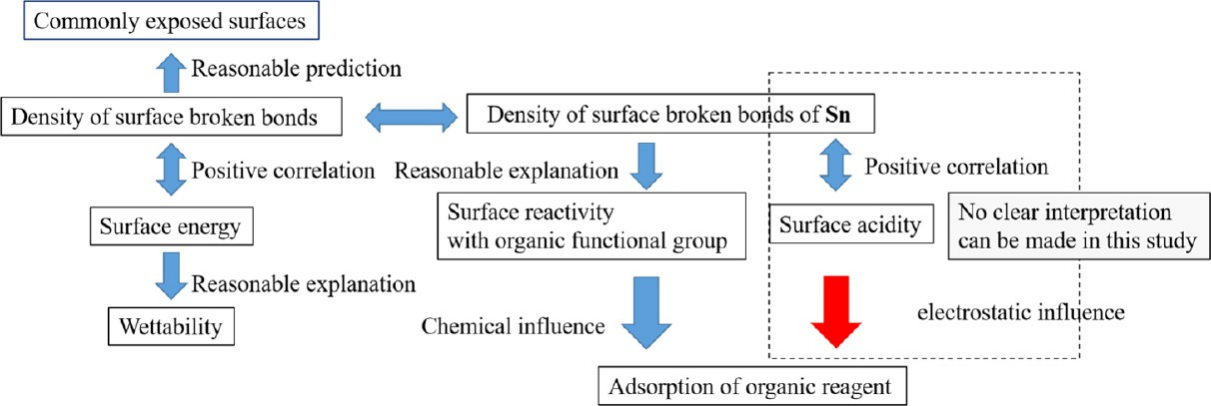
Graphic explanation of the links between the applied concepts.

In summary, as illustrated in Figure 8, the found phenomena of wettability, adsorption difference of surfactant, and the discussed concept surface acidity could be linked with each other based on the ionic density and the density of the broken bonds.

Besides the crystallographic orientation effect, it is assumed that trace elements also have a substantial impact on the surface properties and, therefore, collector adsorption. This is the topic of an ongoing investigation.

## 4 Conclusions

Dissimilar interactions were detected on different-oriented surfaces of cassiterite. As pH decreased from 6.2 to 3.1, the interactions go from repulsive to attractive, followed by the range of SnO_2_(100) < SnO_2_(001) ≈ SnO_2_(110). The most potent attractive force was found to be on the (110) cassiterite surface compared to the (100) and (001) cassiterite surface at lower pH. By fitting the force curves in the DLVO theory framework,^44,45^ anisotropic surface potentials were computed between the three sample surfaces following a similar trend as force interaction. This differential surface potential might be due to the difference in Sn cation density and electron affinity.

Furthermore, it was found that the adsorption of anionic surfactant Aerosol 22 is most effective on SnO_2_(110) followed by SnO_2_(100) and SnO_2_(001) in the concentration range from 10^–6^ to 10^–4^ mol L^–1^. Even though there is still uncertainty for understanding the anisotropic adsorption behavior on the three orientations. The reported experimental finding of the interactions in a broad pH range shall also be helpful to understand other collector systems such as phosphonic acid or hydroxamic acid type collectors, which are applied in higher pH ranges than the sulfosuccinamate collector (Aerosol 22) at pH 3 in this study.

## 5 Materials and Methods

### 5.1 Minerals and Chemicals

A range of SnO_2_ single crystals with the crystallographic orientation of (110), (100), and (001) were used for the contact angle and AFM measurements. These crystals were generously offered by Galazka and co-workers,^46^ who synthesized them by physical vapor transport via recombination of SnO and dioxygen. They were characterized by XRD as well as by high-resolution transmission electron microscopy to validate their orientation and their crystal quality, respectively. In the synthesized crystals, SnO_2_ was the single identified mineralogical phase and no SnO, the main precursor for the growth of SnO_2_, was present. The trace elements, measured by electrothermal vaporization inductively coupled plasma optical emission spectrometry, all occurred in concentrations of less than 10 ppm in the SnO_2_ single crystals. For detailed information about crystal growth and characterizations, please refer to the articles published by Galazka and co-workers.^46,47^

Aerosol 22 (35% tetrasodium *N*-(1,2-dicarboxyethyl)-*N* octadecyl sulfosuccinamate), the collector formulation used for the cassiterite surface functionalization, was supplied by Sigma-Aldrich and used as received. Hydrochloric acid (∼37%), sodium hydroxide (≥99%), potassium chloride (≥99.5%), and ethanol (ROTISOLV HPLC Gradient Grade) were used to adjust the pH, to prepare the background solutions, and to clean the samples, respectively. They were all supplied by Carl Roth GmbH and used as received. DYNASYLAN F8261 (≥97%, Evonik), which comprises a PTFE-like functional group (tridecafluorotriethoxysilan), was used to hydrophobize the colloidal probes.

### 5.2 Sample Cleaning

Prior to all AFM measurements as well as contact angle measurements, the samples were polished with a DiaPro 1/4 μm diamond suspension on a DP-Nap polishing cloth for 30–40 s and subsequently cleaned in a beaker with Milli-Q water (conductivity: 0.055 μs/cm at 25 °C) in an ultrasonic bath for 10 min. For force mapping between silanized silica and hydrophobic surfaces, gas plasma was used after the ultrasonic bath to ensure that organic residuals were removed. However, gas plasma cleaning was not applied for force mapping between silica and SnO_2_ surfaces to avoid the risk of altering the surface charge as it was found that during the cp-AFM-hydrophilic measurements, only large repulsive interactions were detected when plasma treatment was applied in preparation for these measurements. Instead, samples were cleaned in Milli-Q water in an ultrasonic bath for 1 h while changing the Milli-Q water every 10 mins.

### 5.3 Atomic Force Microscopy

The AFM measurements were carried out with an XE-100 (Park Systems), including topographic imaging and roughness quantification, force mapping between silica and bare SnO_2_ surfaces, as well as between silanized silica and SnO_2_ with Aerosol 22 adsorbed on the surfaces. For measurements in an aqueous solution, a liquid probe hand and a PTFE liquid sample containment were used. The samples used for the AFM measurements, representing the three different surfaces, were all embedded in the same epoxy resin block and measured in the same solution with the same colloid sphere. During the AFM measurements, three sites on each sample were randomly chosen and measured separately.

#### 5.3.1 Topographic Imaging and Roughness Quantification

To evaluate the influence of sample polishing as part of the sample preparation procedure, the roughness measurements were repeated 5 times before and after multiple polishing cycles. Surface roughness was measured on sample surface areas of 2 μm × 2 μm as well as 8 μm × 8 μm in tapping mode using an NCHR (non-contact high resolution) cantilever (NanoAndMore GmbH). Topographic imaging of Aerosol 22 adsorptions on the cassiterite surface in the air was also performed in tapping mode with an NCHR cantilever, while in KCl solution, tapping mode was applied using a soft cantilever designed by contact mode ContAl-G (BudgetSensors) with a scan rate of 0.5 Hz. The standard resonance frequency of ContAl-G in the air is 13 kHz; in solution, it decreased to 7.5 kHz.

The topography of the adsorbed Aerosol 22 layers was investigated by AFM. For the topography investigation in air, the sample was removed from the solution and blown dry with air. For the topography investigation in liquid, the sample surface was directly investigated in the Aerosol 22 solution.

#### 5.3.2 Force Mapping and Colloidal Probe Preparation

The colloidal probe cantilevers were prepared by gluing 19.59 μm diameter^48^ spherical silica particles (microparticles GmbH) onto a tipless contact-mode cantilever (image in Appendix). The colloidal probes were prepared using U.V. glue (Ber-Fix Gel), and the silanization of the spherical silica was followed by the steps described by Babel and Rudolph.^49^ Tipless All-in-One B and TL-CONT (Nano and more GmbH) cantilevers were used for the hydrophobic silanized silica particles and silica particles, respectively.

For the force mapping, the spring constant of the cantilever is calibrated by using the bare cantilever without attached sphere measuring the first harmonic vibration frequency ω* (s^–1^) in air. With the given width *w* (m), length *l* (m), thickness *t* (m), and density ρ_cantilever_ (kg m^–3^) of the cantilever, Butt et al.^23^ derive an expression for the spring constant (kg s^–2^)
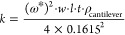
1

The hydrophilic force measurements (cp-AFM-hydrophilic) were conducted in 10 mmol/L KCl solution at pH from 3.1 to 6.2. This pH range was selected since the IEP of stannic oxide (cassiterite) is reported to be around 4.1.^50^ The silica was well known to be negatively charged at the board range of pH (IEP at pH ≈ 2–3^31,33^). Force switching point, where the net interaction between the negatively charged silica and the sample surface changes from attractive to repulsive, would be expected in this pH range. Since the measurement of smaller hydrophilic forces requires higher sensitivity, the movement of the cantilever was thus chosen in a smaller scan area to reduce the fluctuation of the cantilever deflection. The approach distances varied from 600 to 350 nm corresponding to tip velocities of 0.6–0.3 μm s^–1^, respectively. The mapping areas were set to 2 μm × 2 μm with 64 points.

The hydrophobic force measurements (cp-AFM-hydrophobized) were aimed to study the influence of crystallographic orientation on surfactant adsorption. We used the anionic surfactant Aerosol 22 (sulfosuccinamate) to functionalize the different samples at pH 3, which is the most efficient pH value^43^ for Aerosol 22 adsorptions in terms of cassiterite flotability. The mapping areas were set to 8 μm × 8 μm with 64 points. Three sites were chosen for each measurement.

All force curves were measured 10 min after the sample was submerged in solution in a liquid cell with a temperature set to 20 °C. Data evaluation included force normalization, for which the measured forces were divided by the radius of the silica sphere, *R* = 9.75 μm. All four types of cantilevers which were applied in the AFM measurements are summarized in Table 3. The NCHR (NanoAndMore GmbH) coated with aluminum, which displays a spring constant of 42 N/m, was selected for standard topographic imaging since it is traditionally used for this purpose. Meanwhile, the ContAl-G (BudgetSensors), which has a smaller spring constant (0.2 N/m), was reported^51^ to be suitable for scanning soft materials in contact or mapping mode in the liquid phase and therefore was used in our study. Moreover, tipless All-in-one B was chosen for hydrophobic force mapping since it has been successfully employed for this kind of measurement in other studies.^48,52^ Finally, as the hydrophilic interactions are significantly smaller than the hydrophobic forces, we used a TL-CONT cantilever for hydrophilic force mapping, which displays a spring constant of 0.2 N/m, close to that of the cantilever used in the study of rutile surfaces by Bullard and Cima.^15^

### 5.4 Contact Angle Measurements

Contact angle measurements were performed with an electronic dosing system of a commercial contour analysis setup (DataPhysics OCA 50 Pro). Both sessile drop and captive bubble methods were applied. At least eight measurement points were carried out for each sample surface under each measurement condition. For the bare cassiterite surface, the captive bubble method was chosen since the wettability of cassiterite is quite good for a water droplet to spread quickly, which makes the sessile drop method difficult to apply. However, this latter was selected when the surfaces were conditioned with different concentrations of Aerosol 22, as it is significantly less affected by spreading due to the stronger hydrophobicity.

## Figures and Tables

**Figure 9:**
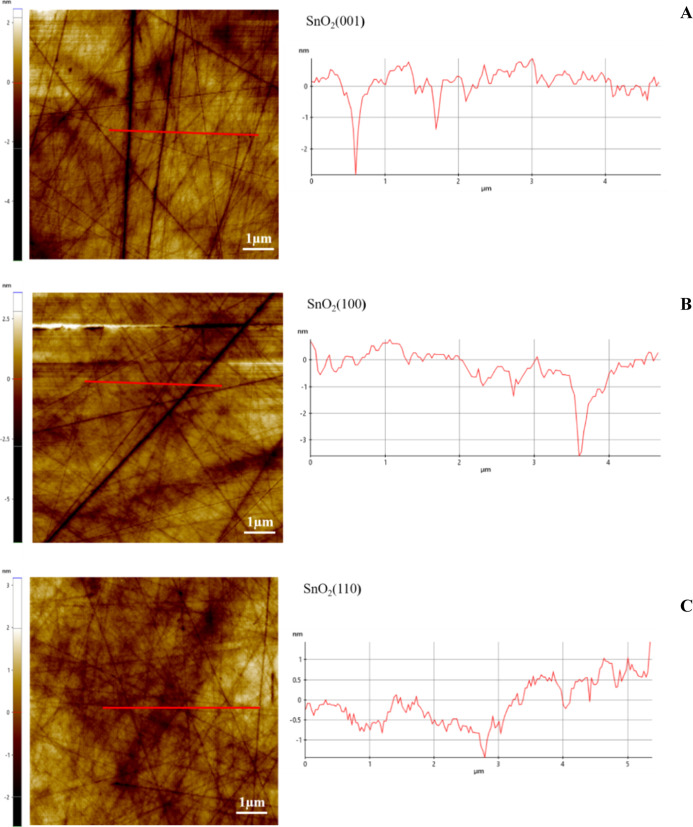
Surface topographies of the polished cassiterite surfaces (A) (001), (B) (100), and (C) (110) orientation measured in tapping mode with a NCHR cantilever. The measuring range is 8 μm × 8 μm.

**Figure 10:**
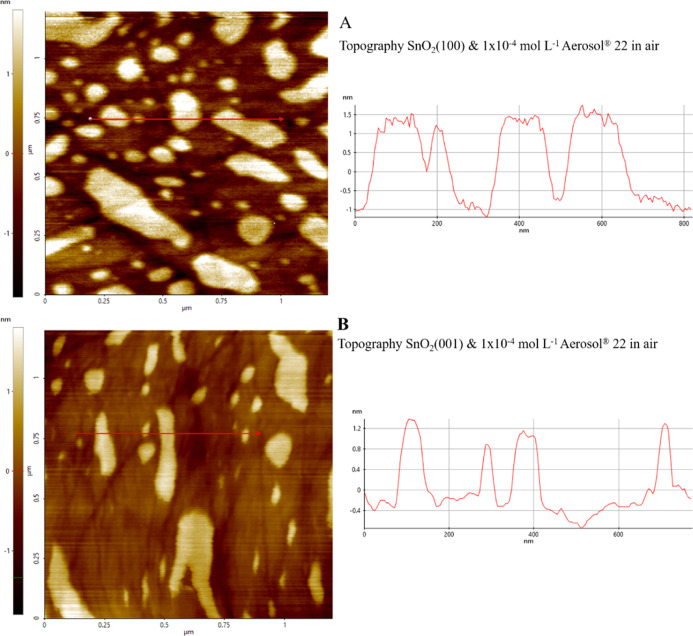
(A) Surface topographies of conditioned (100) cassiterite surface in 1.2 μm × 1.2 μm. (B) Surface topographies of conditioned (001) cassiterite surface in 1.2 μm × 1.2 μm. (A,B) Samples were conditioned with 1 × 10^–4^ mol L^–1^ Aerosol 22 and measured in the air after the sample was blown dry with oxygen (followed the same sample preparation for contact angle measurement).

**Figure 11:**
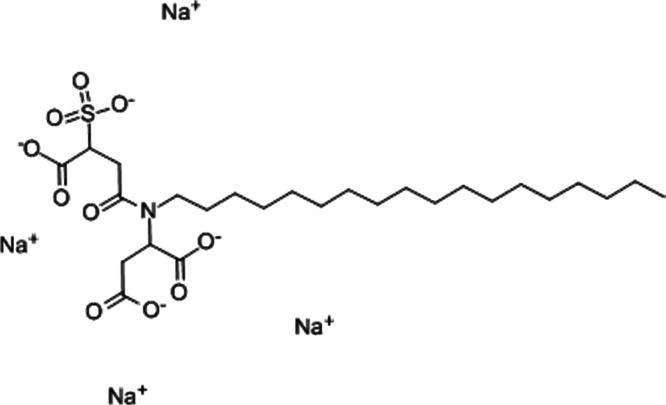
Aerosol 22 structure (CAS no. 38916-42-6, Chemical Book).

**Figure 12:**
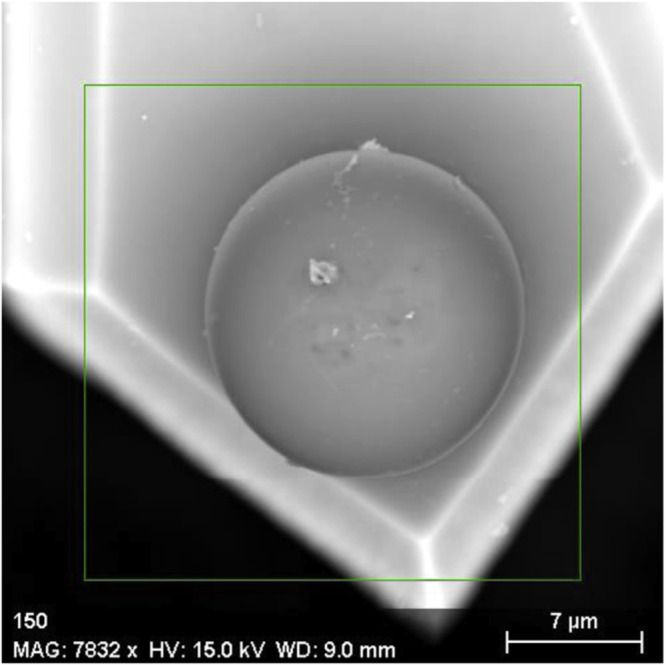
Scanning electron microscopy image of silica glued on the tipless cantilever. This image is reprinted from Babel, B.; Rudolph, M. Fast Preparation and Recycling Method for Colloidal Probe Cantilevers in Hydrophobic Mapping Applications. *MethodsX*. **2019**, *6*, 651.

**Figure 13:**
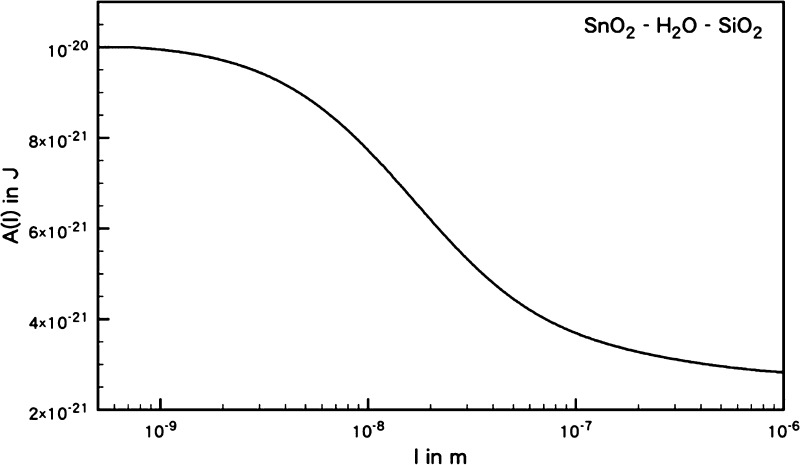
Calculated Hamaker coefficient of the system SnO_2_–H_2_O–SiO_2_ as a function of separation distance.

**Figure 14:**
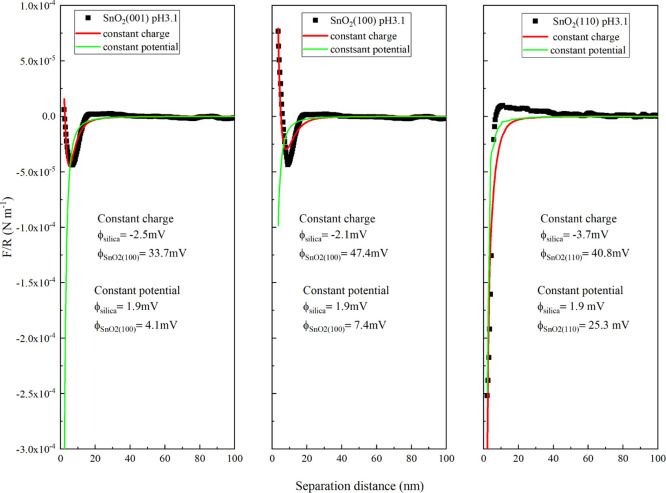
Averaged normalized force curves (*F*/*R*) as a function of the separation distance between silica particles to the sample surface at pH 3.1 with DLVO fitting both under the boundary condition of constant charge and constant potential.

**Figure 15:**
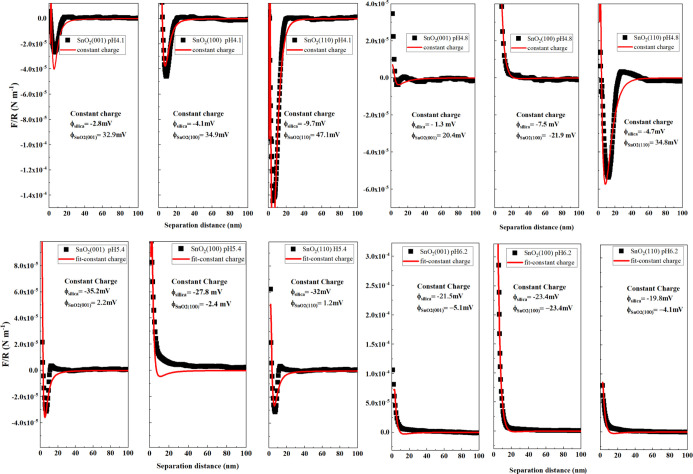
Averaged normalized force curves (*F*/*R*) as a function of the separation distance at pH 4.1, 4.8, 5.4, and 6.2 with DLVO fitting under the boundary condition of constant charge and the corresponding computed surface potentials of silica and sample.

**Figure 16:**
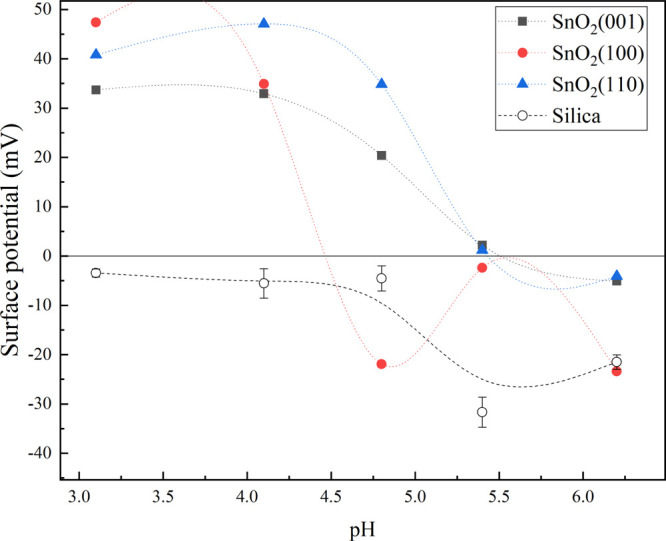
Calculated surface potential of (110), (100), and (001) cassiterite surfaces based on the best results of DLVO fitting at a constant charge as a function of pH as well as the average value of the surface potential of silica for fitting the three sample systems with error bar showing 1 standard deviation.

## Appendix

### Sample Surface Topography

As shown in Figure 9, some visible deeper strips were found at certain places on each sample, which were intentionally avoided during the AFM measurements.

The diamond polishing solution caused the shallow strip shapes on each sample. The depth of the strip varies within a 1 nm range. Roughness measurements before and after multiple polishing cycles show that the surface roughness does not depend on the number of polishing cycles.

### Aerosol 22 Structure

The size of a free molecule was determined by the MolView (molview.org) software, spanning from the methyl-group of the chain to the central nitrogen atom with 2.3 nm and to the most distant oxygen atom in the head group with 2.8 nm (Figure 11).

### Silica Probe

Please see Figure 12.

### Discussions about Extracting Diffuse-Layer Potentials from DLVO Fitting

Within the framework of DLVO,^44,45^ it is assumed that van der Waals and electric double layer forces are operative and that these are additive so that the total force *F* can be decomposed as *F* = *F*_vdW_ + *F*_EDL_.

For separation distances l far smaller than the radius *R* of the colloidal probe, the van der Waals force is calculated^53^ as

1

#### Hamaker Coefficient Calculation

This parameter has been obtained by a numerical solution of the full Lifshitz theory (see, for instance, Adrian Parsegian^54^ for details). The relevant spectral data for SnO_2_ needed for the calculations are presented in Weber et al.^55^ Spectral data for SiO_2_ were taken from Hough and White^56^ and Roth and Lenhoff^57^ for water.

Figure 10 shows the Hamaker coefficient as a function of separation distance. *A*(*l*) is positive throughout the separation distance, indicating an attractive van der Waals interaction, with 1.01 × 10^–20^ J in the zero-separation limit. Retardation reduces its magnitude, starting at about 1 nm.

#### Electric Double-Layer Force Calculation

The electric double-layer force calculation is based on the linearized charge regulation theory reported by Carnie and Chan.^24^ These authors used the linearized form of the Poisson–Boltzmann equation in combination with a linearized charge regulation condition. For the interaction between a sphere and a flat surface, in the Derjaguin approximation, the electric double-layer force can be calculated as

2

with

3

where *T* is the temperature, *R* the ideal gas constant, *F* the Faraday constant, *c* the concentration of the background electrolyte, ε_0_ the permittivity in a vacuum, ε the dielectric constant of the solvent, ψ_*i*_ the diffuse layer potential of surface *i*, and δ_*i*_ is the regulation capacity for the represented surface. A value of δ = 1 denotes the constant potential boundary condition, and δ = −1 corresponds to the constant charge boundary condition. Any value in between accounts for charge regulation.

Because of the use of the linearized Poisson–Boltzmann equation, the formula is valid up to ≈± 50 mV for a 1:1 electrolyte and κ*R″* 1. This latter condition favors the Derjaguin approximation since it works well for large κ*R* and small κ*l*.

#### Two Boundary Conditions and the Understanding of the Silica-Water-Cassiterite Hetero-Interaction System

While the van der Waals component of the total interaction force can often be computed quite reliably in advance, the electric double-layer force calculations are more problematic. This is especially the case when hetero-interactions are considered.

If a double-layer model has been chosen upon which the theory is formulated, the first critical point is choosing the proper boundary condition (i.e., constant charge, constant potential, or charge regulation) for a given system. Without independent experimental input, it is usually challenging to tell which boundary condition is most suitable in advance. Some helpful guidelines for hetero-interaction of diffuse double layers are summarized from Chan et al.:^58^

At a constant charge, they quote: “like charges to be always repulsive” and “unlike charges to be attractive at large separation and repulsive at small separation—except when the surfaces have equal and opposite charges where the interaction is then always attractive.”

At constant potential, “unlike potentials will always attract” and “like (but not identical) potentials will repel at large separations and attract at small separation. Identical surface, however, will always repel.”

The experimental force–distance data of the three cassiterite surfaces at pH 3.1 are shown here in Figure 14. It should be noticed that there is a small repulsive maximum at an intermediate distance on the (110) surface. After the repulsive maximum, the interaction between silica and (110) surface becomes attractive at closer separations, which hints toward a constant potential interaction between surfaces of like sign potentials. On the other hand, the force measured on the (100) and (001) surfaces are both attractive at an intermediate distance; after the attractive maximum, the interaction becomes repulsive, which can be better explained at the constant charge interaction. Furthermore, the fits for the force–distance curves at constant charge are continuously much better than at constant charge for all force curves at the pH of 4.1, 4.8, 5.2, and 6.2 (shown in Figure 15). It is very challenging to explain the detected force–distance relationship measured on the (110) surface at pH 3.1, and it is rather a hint that the surfaces are charge regulating.^59^

Furthermore, a summary of the calculated surface potential of silica and the three samples is also shown in Figure 16. The computed surface potentials of silica at each pH are similar to the reported zeta potentials of silica nanoparticles.^31,32,60^ However, the fitted surface potential for silica varies strongly. The results would thus have a potentially large deviation.

Nevertheless, the surface potential computed from the well-fitted force curve reveals the anisotropic surface potential of the three sample surfaces.

### Data for Two-Sample *T*-Test

Please see Tables 4–8 for the data.

**Table 4:** The Number of Measure Points *N*, Average Mean, Standard Deviation S.D., as well as the Variance of the Normalized Adhesion Force Measured for (110), (100), and (001) Sample Surfaces in a Solution with 1 × 10^–4^ mol L^–1^ Aerosol 22 at pH 3.1

	*N*	mean	SD	variance
SnO_2_(001)	318	–46.40277	8.60634	74.06908
SnO2(100)	200	–47.86818	5.14226	26.44289
SnO2(110)	347	–51.86792	8.09218	65.4834

**Table 5:** *F* Statistics with Calculated *F*-Value, the Numerator Nemer^a^

comparison	*F*-value	numer. DF	denom. DF	prob > *F*
SnO_2_(001) vs SnO2(100)	2.8011	317	199	3.11767 × 10^–14^
SnO_2_(100) vs SnO2(110)	0.40381	199	346	9.57093 × 10^–12^
SnO_2_(001) vs SnO2(110)	1.13111	317	346	0.2619

a D.F., the denominator values of DF denom. *F* as well as the probability for the value larger than *F*. Compared to SnO_2_(001) and SnO_2_(100), at the 95% confidence level, the two population variance is significantly different. Compared to SnO_2_(001) and SnO_2_(110), at the 95% confidence level, the two population variance is significantly different. Compared to SnO_2_(001) and SnO_2_(110), at the 95% confidence level, the two population variance is **NOT** significantly different.

**Table 6:** *T*-Test Compared SnO_2_(001) and SnO_2_(100)^a^

	*t* statistic	DF	prob > |*t*|
**equal variance assumed**	**2.17564**	**516**	**0.03004**
equal variance NOT assumed (Welch correction)	2.4251	514.79367	0.01565

a When the equal variance is assumed at the 95% confidence level, the mean of SnO_2_(001) and SnO_2_(100) is significantly different from 0. When equal variance is not assumed at the 95% confidence level, the mean of SnO_2_(001) and SnO_2_(100) is significantly different from 0.

**Table 7:** *T*-Test Compared SnO_2_(100) and (110)^a^

	*t* statistic	DF	prob > |*t*|
**equal variance assumed**	**6.29454**	**545**	**6.34511 × 10**^**–10**^
equal variance NOT assumed (Welch correction)	7.06039	539.89053	5.13032 × 10^–12^

a When equal variance is assumed at the 95% confidence level, the mean of SnO_2_(100) and (110) is significantly different from 0. When equal variance is not assumed at the 95% confidence level, the mean of SnO_2_(100) and (110) is significantly different from 0.

**Table 8:** *T*-Test Compared SnO_2_(001) and SnO_2_(110)^a^

	*t* statistic	DF	prob > |*t*|
equal variance assumed	8.43919	663	2.00495 × 10^–16^
**equal variance NOT assumed** (Welch correction)	**8.41655**	**648.65076**	**2.47913 × 10**^**–16**^

a At the 95% confidence level, when the equal variance is assumed, the mean of SnO_2_(001) and SnO_2_(110) is significantly different from 0. At the 95% confidence level, when the equal variance is NOT assumed, the mean of SnO_2_(001) and SnO_2_(110) is significantly different from 0.
